# Selective innervation of NK1 receptor–lacking lamina I spinoparabrachial neurons by presumed nonpeptidergic Aδ nociceptors in the rat

**DOI:** 10.1016/j.pain.2014.08.023

**Published:** 2014-11

**Authors:** Najma Baseer, Abdullah S. Al-Baloushi, Masahiko Watanabe, Safa A.S. Shehab, Andrew J. Todd

**Affiliations:** a Institute of Neuroscience and Psychology, College of Medical, Veterinary, and Life Sciences, University of Glasgow, Glasgow, UK; b Department of Anatomy, College of Medicine and Health Sciences, United Arab Emirates University, Al Ain, United Arab Emirates; c Department of Anatomy, Hokkaido University School of Medicine, Sapporo, Japan

**Keywords:** Cholera toxin B subunit, Dorsal horn, Lamina I projection neuron, Spinal cord, Transganglionic tracing

## Abstract

Fine myelinated (Aδ) nociceptors are responsible for fast, well-localised pain, but relatively little is known about their postsynaptic targets in the spinal cord, and therefore about their roles in the neuronal circuits that process nociceptive information. Here we show that transganglionically transported cholera toxin B subunit (CTb) labels a distinct set of afferents in lamina I that are likely to correspond to Aδ nociceptors, and that most of these lack neuropeptides. The vast majority of lamina I projection neurons can be retrogradely labelled from the lateral parabrachial area, and these can be divided into 2 major groups based on expression of the neurokinin 1 receptor (NK1r). We show that CTb-labelled afferents form contacts on 43% of the spinoparabrachial lamina I neurons that lack the NK1r, but on a significantly smaller proportion (26%) of those that express the receptor. We also confirm with electron microscopy that these contacts are associated with synapses. Among the spinoparabrachial neurons that received contacts from CTb-labelled axons, contact density was considerably higher on NK1r-lacking cells than on those with the NK1r. By comparing the density of CTb contacts with those from other types of glutamatergic bouton, we estimate that nonpeptidergic Aδ nociceptors may provide over half of the excitatory synapses on some NK1r-lacking spinoparabrachial cells. These results provide further evidence that synaptic inputs to dorsal horn projection neurons are organised in a specific way. Taken together with previous studies, they suggest that both NK1r^+^ and NK1r-lacking lamina I projection neurons are directly innervated by Aδ nociceptive afferents.

## Summary

This study provides evidence that nonpeptidergic Aδ nociceptive primary afferents selectively innervate lamina I spinoparabrachial projection neurons that lack the neurokinin 1 receptor.

## 1 Introduction

Myelinated nociceptive primary afferents, most of which conduct in the Aδ range, convey information that is perceived as fast pain [41]. Although some myelinated (Aβ/Aδ) nociceptors have axons that extend throughout laminae I–V, many Aδ nociceptors arborise mainly in lamina I of the dorsal horn [29], [67], [68], a region that contains a high density of projection neurons, nearly all of which can be retrogradely labelled from the lateral parabrachial area [1], [38], [57].

Although virtually all lamina I projection neurons in rat respond to noxious stimuli [3], [7], [21], anatomical studies have identified specific populations that differ in their synaptic inputs. The majority (75% to 80%) of the projection cells express the neurokinin 1 receptor (NK1r), and these cells are densely innervated by peptidergic primary afferents, which are thought to provide approximately half of their excitatory synapses [36], [60]. We have identified a small but distinctive population of giant projection neurons in lamina I that lack the NK1r. These cells are densely innervated by both excitatory and inhibitory interneurons, but seem to receive little (if any) direct primary afferent input [35], [40]. Virtually nothing is known about the synaptic inputs to the remaining (NK1r-lacking) projection neurons in this lamina, although it has been shown that they receive a lower density of contacts from peptidergic primary afferents than the NK1r^+^ projection neurons [60]. It also has been reported that some of these cells can express NK1r de novo after peripheral nerve injury [47].

Cholera toxin B subunit (CTb) binds to the GM1 ganglioside, and when injected into intact somatic peripheral nerves, it is taken up and transported mainly by myelinated primary afferents. This results in labelling of axonal boutons in lamina I and in a region of the dorsal horn that extends ventrally from the inner half of lamina II (IIi) [17], [23], [43], [49], [69]. The labelling in lamina I is thought to correspond to central terminals of myelinated nociceptors, in particular those with Aδ axons, whereas that in deeper laminae is mainly in low-threshold mechanoreceptive Aδ and Aβ afferents [23], [29], [43].

Lamina I projection neurons are known to respond to activity in Aδ afferents [7], and it has been reported that for at least some cells this is mediated through monosynaptic inputs [4], [63], [64]. However, the postsynaptic targets of CTb-labelled (presumed myelinated nociceptive) afferents in lamina I apparently have not been identified. The main aim of this study therefore was to test the hypothesis that these afferents are presynaptic to projection neurons in this lamina, and to determine whether such inputs preferentially target specific types of projection cell. Some Aδ nociceptors express the neuropeptides calcitonin gene-related peptide (CGRP) and substance P, and these differ in their receptive field properties from Aδ nociceptors that lack these peptides [25], [26]. However, a preliminary immunofluorescence study suggested that there was little or no transport of CTb by peptidergic afferents [42]. We therefore tested whether CGRP or substance P were present in CTb-labelled boutons in lamina I and related this to expression of the vesicular glutamate transporter VGLUT2, which has been found in approximately 80% of these boutons [58].

## 2 Methods

### 2.1 Animals and tissue processing

All experiments were approved by the Animal Ethics Committee of the College of Medicine and Health Science of the United Arab Emirates University and were performed in accordance with the guidelines of the European Communities Council directive of 24 November 1986 (86/609/EEC).

Nine adult male Wistar rats (240 to 255 g; UAE University) were used in this study. All animals were anaesthetised with ketamine and xylazine (25 mg and 5 mg intramuscularly, respectively) and received an injection of 2 μL of 1% or 2% CTb into the left sciatic nerve, as described previously [51]. Six of the rats were placed in a stereotaxic frame immediately after the nerve injection, and these animals received an injection of 50 nl 4% Fluorogold targeted on the lateral parabrachial area (LPb), to label spinoparabrachial neurons [59]. Because the majority of lamina I spinoparabrachial neurons project contralaterally, the Fluorogold injection was targeted on the LPb on the right side. The animals made an uneventful recovery from general anaesthesia. Three days after the injections, they were terminally anaesthetised with urethane (625 mg intraperitoneally) and perfused with fixative containing 4% freshly depolymerised formaldehyde through the left cardiac ventricle. The L4 spinal segments of all rats and the brains of those that had received stereotaxic injections were removed and post-fixed for 4 hours. The brains were cut into 100-μm coronal sections with a freezing microtome, and these were used to assess the spread of Fluorogold. The spinal cord segments were cut into transverse or horizontal sections (60 μm thick) with a vibrating microtome and processed as described later.

### 2.2 Neurochemical analysis of CTb-labelled boutons in lamina I

Transverse spinal cord sections from the 3 rats that received only sciatic nerve injections were incubated for 3 days with goat anti-CTb, rabbit anti-VGLUT2, rat anti-substance P, and guinea pig anti-CGRP. Details of the sources and dilutions of the primary antibodies are given in Table 1. The sections were then incubated overnight in species-specific secondary antibodies raised in donkey and conjugated to Alexa 488 (Life Technologies) or to Rhodamine Red, DyLight 649, or biotin (Jackson Immunoresearch). All secondary antibodies were diluted 1:500, apart from those conjugated to Rhodamine Red, which were diluted 1:100. The biotinylated antibody was revealed with Pacific Blue conjugated to avidin (1:1000; Life Technologies). Sections were mounted in antifade medium and stored at −20°C. All antibodies used in this part of the study were diluted in phosphate-buffered saline that contained 0.3% Triton X-100, and incubations were at 4°C.

**Table 1:** Primary antibodies used in this study.

Antibody	Species	Catalogue number	Dilution	Source
CTb	Goat	703	1:5000	List biological
CTb	Mouse	ab35988	1:5000	Abcam
VGLUT2	Goat		1:500	M. Watanabe
VGLUT2	Rabbit	135 402	1:5000	Synaptic systems
CGRP	Guinea pig	T-5027	1:10,000	Bachem
Substance P	Rat	OBT06435	1:200	Oxford biotech
NK1r	Rabbit	S8305	1:10,000	Sigma Aldrich
Fluorogold	Guinea pig	NM101	1:500	Protos biotech

Three sections were selected from each rat and scanned with a Zeiss LSM 710 confocal microscope (with Argon multiline, 405 nm diode, 561 nm solid-state and 633 nm HeNe lasers) through a 63× oil-immersion lens (NA 1.4) and a pinhole of 1 Airy unit. Several overlapping z-stacks (20 optical sections at 0.5 μm z-separation) were scanned so as to include the whole of lamina I on the left side. Sections were analysed with Neurolucida for Confocal software (MicroBrightField), and from each section, 100 CTb-immunoreactive boutons were selected from across the full mediolateral extent of CTb labelling in lamina I. This selection was made before other channels were viewed. The remaining channels were then switched on and the neurochemical phenotype of each of the selected CTb boutons was assessed. In order to determine whether variations in the sizes of different types of bouton could have resulted in a sampling bias, we measured the z-axis lengths of a sample of boutons of each of the major neurochemical types by determining the number of optical sections on which they appeared and multiplying this by 0.5 μm (the z-spacing) [48].

### 2.3 Contacts between CTb-labelled Aδ afferents and lamina I spinoparabrachial neurons

Horizontal sections of spinal cord from 4 of the rats that received sciatic and LPb injections were incubated in mouse anti-CTb, rabbit anti-NK1r, goat anti-VGLUT2, and guinea pig anti-Fluorogold, which were revealed with fluorescent secondary antibodies as described earlier. In order to estimate the proportion of projection neurons with or without the NK1r that received contacts from CTb-labelled Aδ afferents, 1 or 2 horizontal sections that contained the largest number of lamina I projection neurons were selected from each rat and scanned through the 40× objective lens (NA 1.3) with a z-step of 1 μm. A set of overlapping fields was scanned to include the entire mediolateral and rostrocaudal extent of lamina I on the left side within each of these sections. All retrogradely labelled cells, apart from those that had substantial parts of the soma or dendritic tree missing from the section, were identified and classified into 1 of 3 types: (1) NK1r-immunoreactive cells (NK1r^+^), (2) giant cells (identified by the high density of VGLUT2 boutons on their cell bodies and proximal dendrites [35]), and (3) projection neurons that were not giant cells and that lacked the NK1r (NK1r-lacking cells). For each cell, the dendritic tree was followed as far as possible through the z-stack and the presence or absence of contacts from CTb-labelled boutons was recorded.

Examination of these sections revealed that relatively few NK1r^+^ projection cells received contacts from CTb boutons, and these contacts were generally at a low density. In contrast, a higher proportion of NK1r-lacking projection cells were contacted by CTb boutons, and these contacts were more numerous. We therefore carried out a detailed analysis of contact density on a sample of NK1r-lacking and NK1r^+^ projection neurons. Because the degree of CTb labelling can vary among experiments, presumably reflecting differences in the numbers of axons that have taken up the injected tracer, we did not use a random sampling approach to select projection neurons for this analysis. Instead, we selected 20 Fluorogold-labelled NK1r-lacking lamina I cells (4 to 6 from each rat) and 18 Fluorogold-labelled NK1r^+^ cells (3 to 8 cells from each rat) that were seen to receive relatively high numbers of contacts, and scanned the cell bodies and as much of the dendritic tree as was visible within the section. The scans were obtained through the 63× oil-immersion lens to generate z-stacks with a z-separation of 0.5 μm. For both populations of cells, we used Neurolucida for Confocal to plot the locations of boutons in contact with the soma and dendrites of each cell that were CTb- and/or VGLUT2-immunoreactive. Because we found that a few CTb-labelled boutons in lamina I were CGRP-immunoreactive (see Results), we then re-incubated the sections with guinea pig anti-CGRP, revealed this with Pacific blue (the same fluorochrome as had been used to reveal Fluorogold), and rescanned the cells, as described earlier. Although both Fluorogold and CGRP were now labelled with Pacific blue, they could easily be discriminated by comparison with the initial scans, which did not show CGRP immunoreactivity [6]. All CTb boutons contacting the cells were re-examined to determine whether or not they contained CGRP. In addition, the locations of contacts that the cells received from CGRP-immunoreactive boutons that lacked CTb were plotted. Cell body surface areas were measured, and the surface areas of dendrites were estimated from their lengths and diameters, based on the assumption that they were cylindrical [6], [60]. For all types of contact, the density per 1000 μm^2^ of combined somatic and dendritic surface was determined.

### 2.4 Combined confocal and electron microscopy

To confirm that contacts between CTb-labelled afferents and spinoparabrachial neurons were associated with synapses, we used a combined confocal and electron microscopic technique [6], [33], [56]. In preliminary studies we found that CTb was highly sensitive to glutaraldehyde fixation, and in particular CTb immunoreactivity in lamina I was not detected even with very low concentrations of glutaraldehyde in the primary fixative. For this reason we carried out this part of the study on tissue from animals that had been perfusion-fixed with 4% formaldehyde (ie, without glutaraldehyde).

Horizontal sections of spinal cord from 2 rats that had received injections of CTb into the sciatic nerve and Fluorogold into the contralateral LPb were reacted with goat anti-CTb, rabbit anti-NK1r, and guinea pig anti-Fluorogold. The reaction was performed as described earlier, except that: (a) Triton was omitted, (b) the secondary antibody cocktail contained both biotinylated and fluorescent-labelled anti-goat antibodies, and (c) the sections were incubated in avidin conjugated to horseradish peroxidase (HRP; 1:1000; Sigma) before being mounted and scanned [6], [33], [35].

Three retrogradely labelled neurons on the left side (2 NK1r-lacking cells from different animals, and 1 NK1r^+^ cell) that received contacts from CTb axons were selected and scanned. Low magnification confocal z-stacks were obtained to allow subsequent identification of the cells during the preparation of tissue for electron microscopy. High-magnification z-series were scanned with the 63× oil-immersion objective (z-step 0.5 μm) through the cell bodies and dendritic trees of each of these cells, and the locations of contacts that the cells received from CTb-immunoreactive boutons were noted. The sections containing these cells were further fixed with 1% glutaraldehyde in PB overnight. They were then reacted with 3,3′-diaminobenzidine (DAB) in the presence of hydrogen peroxide [6], [33], [35] to reveal CTb. The sections were osmicated, block-stained with uranyl acetate, and embedded in resin. The regions containing the selected cells were identified from the distribution of DAB, which could be correlated with the CTb immunoreactivity seen in the confocal images. The sections were mounted on blocks of cured resin and trimmed to the appropriate area. Series of ultrathin sections were cut through the cells with a diamond knife and collected in serial order on Formvar-coated single-slot grids. These were contrasted with lead citrate and viewed with a Philips CM 100 electron microscope. CTb-immunoreactive boutons could be identified by their DAB reaction product, whereas the dendrites of the selected projection neurons were recognised by their position in relation to these boutons.

### 2.5 Antibody characterisation

Specificity of the CTb and Fluorogold antibodies is shown by lack of staining in regions that did not contain transported tracer. The goat and rabbit VGLUT2 antibodies were raised against amino acids 550 to 582 of rat VGLUT2 and 510 to 582 of mouse VGLUT2, respectively, and both recognise a single protein band of the appropriate molecular weight [20], [55]. The CGRP antibody detects both α and β forms of the peptide. The monoclonal substance P antibody detects the C-terminal 5 to 8 amino acids of the peptide [10], and does not seem to recognise neurokinin B [37]. The NK1r antibody, raised against amino acids 393–407 of the rat NK1r, recognises a 46 kDa band in Western blots of rat brain extracts, and it has been shown that there is no staining with this antibody in mice in which the NK1r has been deleted [39].

### 2.6 Statistics

One-way ANOVA was used to test for differences in the z-axis lengths of different neurochemical types of CTb-labelled bouton in lamina I. A Student *t* test was used to determine whether there was a significant difference in the proportions of NK1r^+^ and NK1r-lacking projection neurons that received contacts from CTb-labelled boutons. Mann-Whitney *U* tests were used to compare densities of contacts from different types of axonal bouton onto these 2 different populations of projection neurons.

## 3 Results

### 3.1 VGLUT2 and neuropeptide expression by CTb boutons in lamina I

**Fig. 1:**
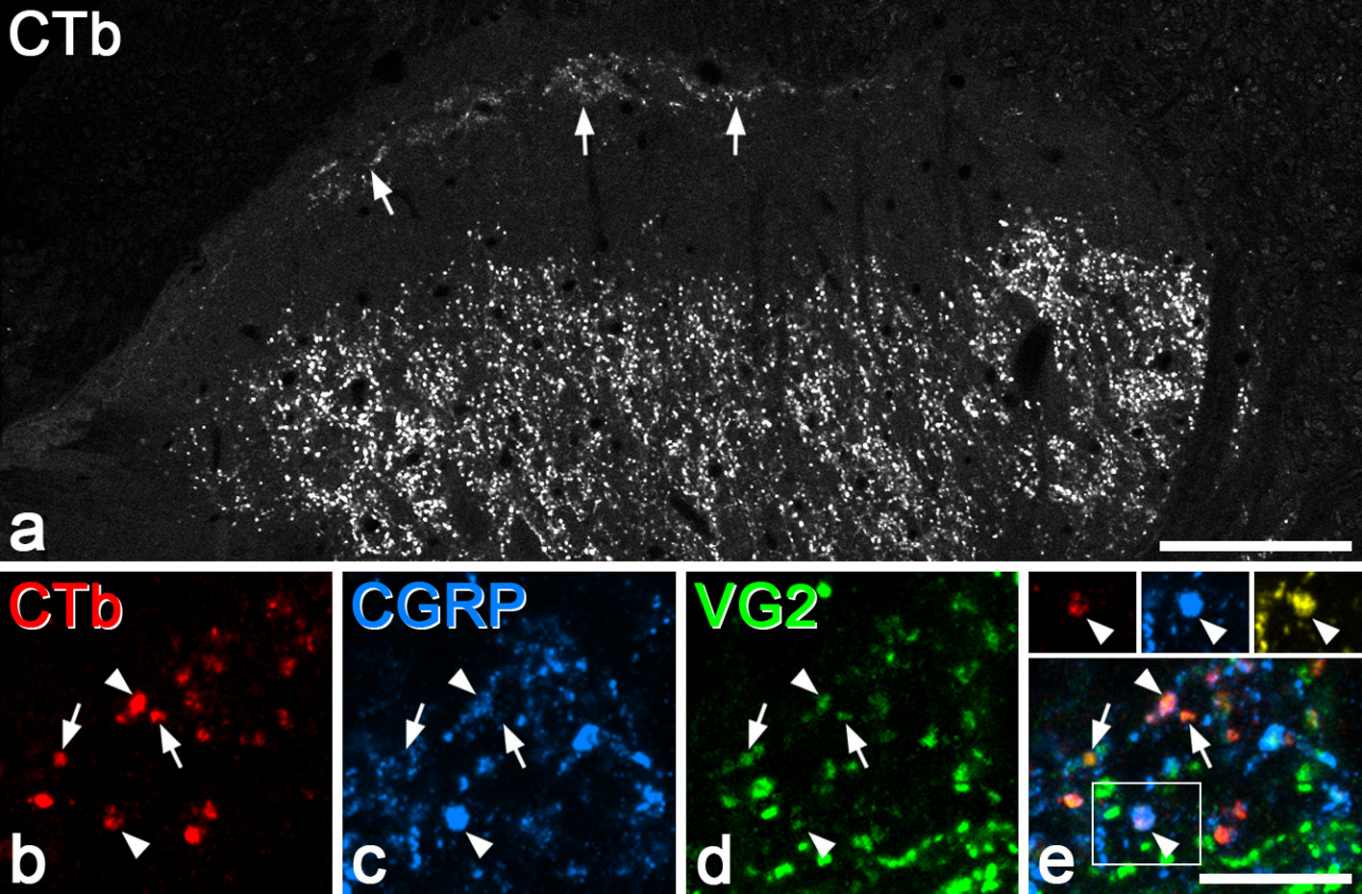
Expression of neuropeptides and VGLUT2 by CTb-labelled primary afferents in lamina I. (a) Low-magnification view of the upper part of the dorsal horn showing the general distribution of CTb-labelled profiles seen in a transverse section. Arrows point to the plexus of labelled axons in lamina I, and below this there are very few labelled structures in the outer part of lamina II. (b–d) Confocal images from a section scanned to reveal (b) CTb (red), (c) CGRP (blue), and (d) VGLUT2 (VG2, green). A merged image is shown (e). Several CTb-immunoreactive boutons are visible. Two of these are CGRP^+^, and these are marked with arrowheads. Arrows indicate 2 CTb-immunoreactive boutons that lack CGRP. Although most of the CTb-labelled boutons in this field contain VGLUT2, the level of expression of the transporter varies considerably between boutons. The inset in (e) (corresponding to the area in the box) shows the lower of the 2 CTb-labelled boutons that are marked with an arrowhead. This has been scanned to reveal CTb (red), CGRP (blue), and substance P (yellow), and the bouton can be seen to contain both peptides. (a) Projection of 2 optical sections at 1 μm z-separation. (b–e) Projection of 2 optical sections at 0.5 μm z-separation. Scale bars: (a) = 100 μm, (b–e) = 10 μm.

After injection of CTb into the sciatic nerve, CTb-immunoreactive boutons were densely distributed throughout the sciatic territory in the deep part of the dorsal horn, extending ventrally from lamina IIi, and in addition there was a sparser plexus of labelled boutons in lamina I in the corresponding region, as described in several previous studies [23], [43], [49], [50], [51], [58], [69] (Fig. 1a). The distribution of staining for VGLUT2, CGRP, and substance P was the same as that described previously [2], [15], [16], [24], [34], [58], and in all cases immunostaining was detected throughout the full thickness of the sections.

Consistent with our previous report [58], we found that the majority of CTb-labelled boutons in lamina I (mean 75%; Table 2) were VGLUT2-immunoreactive, although the strength of immunostaining varied considerably between boutons. Because some Aδ nociceptors are peptidergic [25], [26], and many peptidergic primary afferent terminals in the dorsal horn do not have detectable levels of VGLUT2 [24], [32], [58], we tested whether the CTb^+^/VGLUT2^−^ boutons in lamina I corresponded to peptidergic terminals. Although numerous boutons containing CGRP were observed in lamina I, only 11.4% of the CTb boutons in this lamina showed CGRP immunoreactivity, and most of these (75.9%) were also VGLUT2^+^ (Table 2, Fig. 1b to e). The remaining 24.1% of CGRP^+^ boutons (ie, those that lacked VGLUT2) constituted 2.7% of all CTb-labelled boutons (24.1% of 11.4%), and therefore accounted for only about 10% of the CTb boutons that lacked VGLUT2. Substance P was found in an even lower proportion of CTb-labelled boutons (mean 2.3%), and all of these were CGRP-immunoreactive (Fig. 1e inset). The mean z-axis lengths of nonpeptidergic CTb boutons with and without VGLUT2 was 2.72 ± 0.65 μm and 2.69 ± 0.54 μm, respectively (n = 40 boutons in each case). The corresponding values for CTb boutons with CGRP but not substance P and for those with both peptides were 2.68 ± 0.58 μm and 2.71 ± 0.56 μm, respectively (n = 20 boutons in each case). These values did not differ significantly (ANOVA, *P* = 0.99), indicating that our estimates of the proportion of each neurochemical type are unlikely to have been affected by sampling bias.

**Table 2:** Neurochemistry of CTb boutons in lamina I.

Rat	VGLUT2^+^	CGRP^+^	SP^+^	VGLUT2^+^/CGRP^+^	CGRP^+^ with VGLUT2
1	74	13.3	3.7	9.3	70
2	78.7	9.7	1.7	9	93.1
3	72.3	11.3	1.7	7.3	64.7
Mean	75	11.4	2.3	8.6	75.9

The second to fifth columns show the percentages of lamina I CTb boutons in each of the 3 rats that were immunoreactive for VGLUT2, CGRP, substance P, or both VGLUT2 and CGRP. The sixth column shows the percentage of CGRP^+^ CTb boutons that were also VGLUT2 immunoreactive.

These results indicate that peptidergic afferents (particularly those that contain substance P) are seldom CTb-labelled, and can only account for a small proportion of the CTb^+^/VGLUT2^−^ boutons in lamina I. The discrepancy between our findings and those of Rivero-Melian et al. [42], who did not observe any colocalisation of CTb with either CGRP or substance P, is probably explained by our use of confocal microscopy. This allows more accurate resolution of small profiles within a dense plexus of immunoreactive axons, as well as the detection of weakly labelled boutons.

### 3.2 Contacts between CTb boutons and lamina I projection neurons

**Fig. 2:**
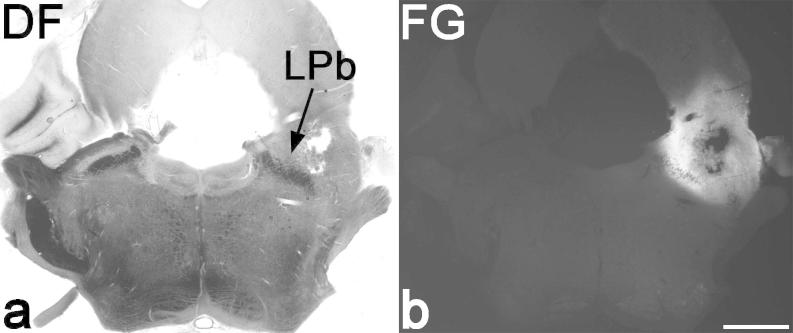
Example of a Fluorogold injection site. (a, b) Dark field (DF) transmitted and fluorescent views of a section through the brainstem of one of the rats used in this study. The Fluorogold (FG) has spread throughout the entire lateral parabrachial area (LPb), with some extension into nearby structures. Scale bar = 1 mm.

In all cases, the Fluorogold injection site included the whole of the LPb, with variable spread into surrounding areas. An example is shown in Fig. 2. Quantitative data from the sections in which Fluorogold-labelled lamina I projection neurons were assessed for the presence or absence of contacts from CTb-immunoreactive primary afferent boutons are shown in Table 3. The mean number of projection neurons identified in each rat was 208, of which 71.6% were NK1r^+^, 2.1% were giant cells, and 26.3% were NK1r-lacking non-giant cells (defined as NK1r-lacking cells for convenience). As reported previously [35], we found that the giant cells did not receive contacts from CTb boutons. In contrast, 26.1% of the NK1r^+^ cells and 43.1% of the NK1r-lacking cells were found to receive contacts from CTb-labelled boutons. The proportions of neurons in these 2 populations that received contacts were significantly different (*P* < .001, *t* test).

**Table 3:** Analysis of contacts onto different types of projection neuron.

Rat	Total projection cells	NK1r^+^ cells	Giant cells	NK1r-lacking cells	NK1r^+^ cells with contacts (%)	NK1r-lacking cells with contacts (%)
1	254	183 (72%)	7 (2.8%)	64 (25.2%)	28	40.6
2	190	134 (70.5%)	3 (1.6%)	53 (27.9%)	26.9	47.2
3	200	128 (64%)	5 (2.5%)	67 (33.5%)	26.6	44.8
4	187	149 (80%)	3 (1.6%)	35 (18.7%)	22.8	40
Mean	207.8	148.5 (71.6%)	4.5 (2.1%)	54.8 (26.3%)	26.1	43.1

The second to fifth columns show the total numbers of projection neurons sampled and the number (percent) belonging to each class for the 4 rats from which sections were analysed. The last 2 columns show the percentage of cells of the corresponding type that received contacts from CTb-labelled boutons. No contacts were seen on the giant cells.

**Fig. 3:**
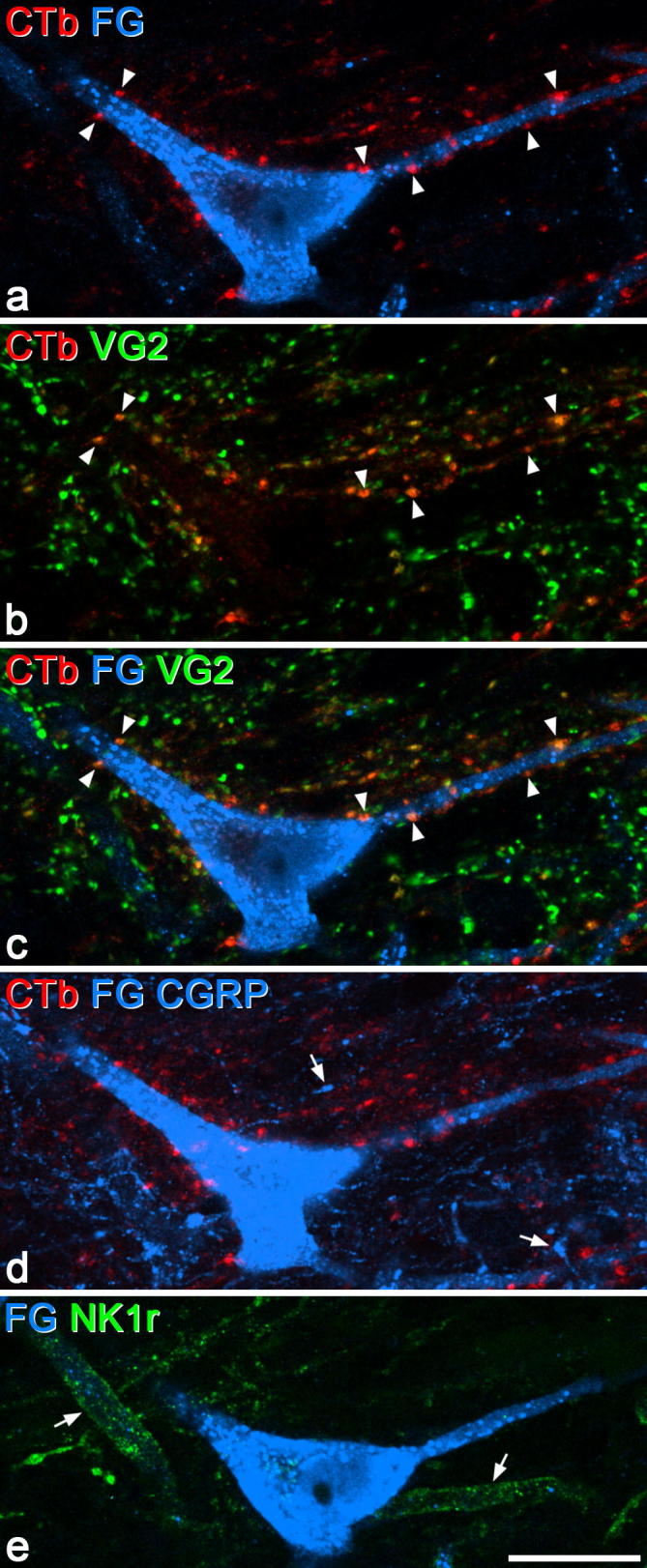
A NK1r-lacking lamina I spinoparabrachial neuron that receives numerous contacts from CTb-labelled primary afferent boutons. Confocal images from a section reacted to reveal Fluorogold (FG), CTb, VGLUT2 (VG2), and the NK1r. (a–c) Projection of 3 optical sections at 0.5 μm z-separation through the cell body and proximal dendrites of a lamina I neuron retrogradely labelled with Fluorogold (blue), which receives numerous contacts from CTb-immunoreactive boutons (red), some of which are indicated with arrowheads. Several of these boutons are also labelled with the VGLUT2 antibody (green), and therefore appear orange in the merged images. The lamina I neuron received relatively few contacts from other VGLUT2^+^ boutons. (d) An equivalent projected image through the same section after it had been reacted to reveal CGRP (which appears in the same colour channel as Fluorogold). CGRP is represented by blue profiles that were not visible in (a), and 2 of these are indicated with arrows. Note that because the section was remounted before scanning, its orientation is not exactly the same as in (a–c), and therefore not all of the CTb-labelled profiles are visible. However, it is clear that none of those contacting the cell are CGRP-immunoreactive. (e) Projection of 3 different z-sections through the same cell to show the relationship between NK1r (green) and Fluorogold (blue). The retrogradely labelled cell lacks the receptor, but there are 2 nearby dendrites that are NK1r^+^ (arrows). Scale bar = 20 μm.

**Fig. 4:**
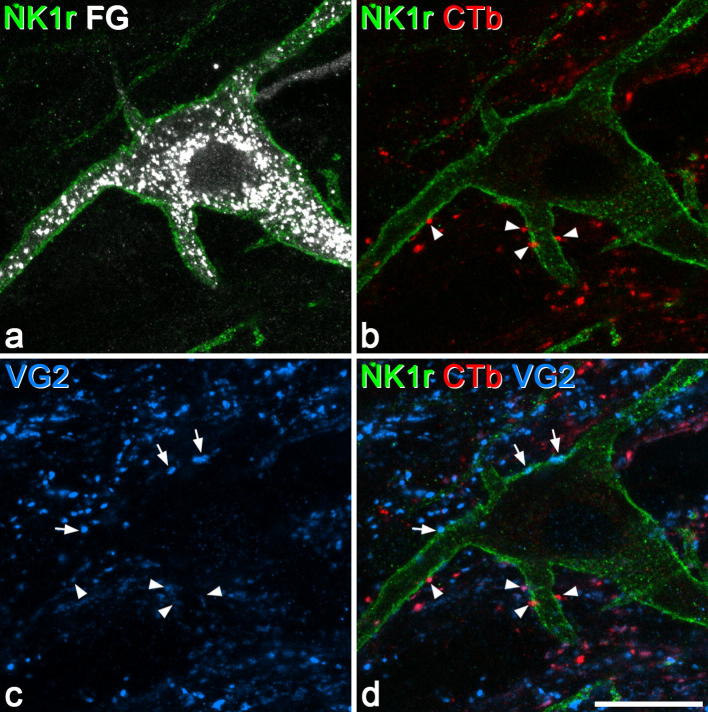
A NK1r^+^ lamina I spinoparabrachial neuron that receives a few contacts from CTb-labelled boutons. (a–d) Combinations of staining for Fluorogold (FG, white), NK1r (green), CTb (red), and VGLUT2 (VG2, blue) in a projection of 6 optical sections (0.5 μm z-separation) through the cell body and proximal dendrites of a lamina I neuron labelled with Fluorogold from the lateral parabrachial area. The cell receives 4 contacts from CTb-labelled boutons (arrowheads), which show weak VGLUT2 immunoreactivity. It also receives several other contacts from VGLUT2-immunoreactive boutons, 3 of which are indicated with arrows. Scale bar = 20 μm.

**Fig. 5:**
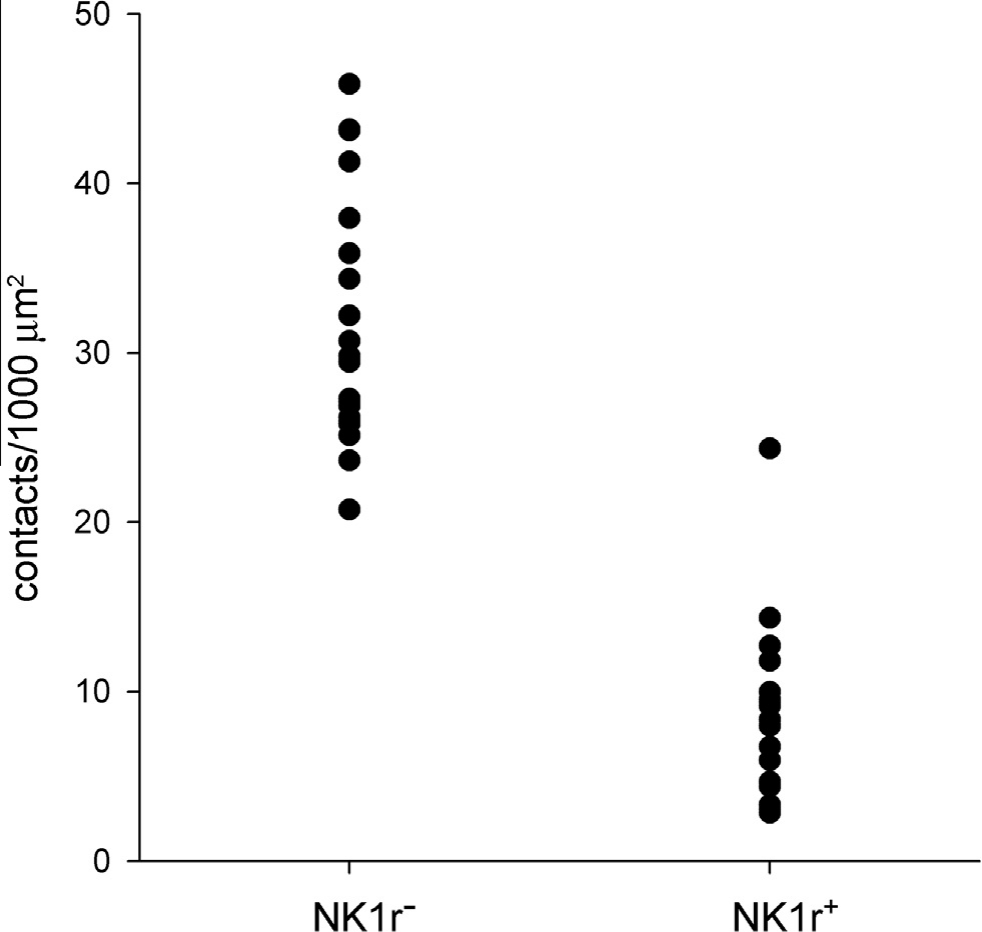
Density of contacts from nonpeptidergic CTb-labelled boutons on projection neurons. A plot of the contact densities for CTb^+^ boutons that lacked CGRP onto projection neurons without (NK1r^−^, n = 20) and with (NK1r^+^, n = 18) the NK1 receptor. Density is expressed as the number of contacts from CTb^+^/CGRP^−^ boutons per 1000 μm^2^ combined somatic and dendritic surface area.

During this part of the study, we observed that those NK1r-lacking projection cells that received contacts from CTb-labelled boutons invariably had several such contacts, whereas for the NK1r^+^ projection cells contacted by CTb boutons, these contacts seemed to be far less numerous. This observation was confirmed by the quantitative analysis of contacts onto the 20 NK1r-lacking cells and 18 NK1r^+^ cells (Table 4, Fig. 3, Fig. 4). The NK1r-lacking projection cells in this sample received a far higher density of contacts from CTb^+^ boutons (31.7/1000 μm^2^) than the NK1r^+^ projection cells (9.1/1000 μm^2^) (*P* < .001, Mann-Whitney *U* test). Virtually all (99.7%) of the CTb^+^ boutons in contact with the NK1r-lacking cells did not contain CGRP, whereas 7% of the CTb boutons that contacted the NK1r^+^ cells were CGRP-immunoreactive. However, the latter accounted for only 2% of all the CGRP boutons in contact with the NK1r^+^ cells. The contact densities for the CTb boutons that lacked CGRP are shown in Fig. 5, and this indicates that virtually all of the NK1r^+^ cells had a lower density in comparison to the NK1r-lacking cells.

**Table 4:** Contact density on NK1r^+^ and NK1r-lacking projection neurons.

	CTb^+^/CGRP^−^		CTb^+^/CGRP^+^		CTb^−^/CGRP^+^		CTb^−^/VGLUT2^+^/CGRP^−^	
	Number	Density	Number	Density	Number	Density	Number	Density
NK1r^+^ (n = 18)	43.6 (14–145)	8.5 (2.8–24.3)	3.2 (0–7)	0.6 (0–1.5)	140.7 (62–266)	26.6 (16.7–43.1)	87.7 (23–137)	16.8 (9.3–31.8)
NK1r-lacking (n = 20)	79.9 (30–210)	31.6 (20.8–45.9)	0.2 (0–2)	0.1 (0–0.5)	7.5 (2–15)	3.1 (1.2–8)	26.5 (5–99)	9.7 (4–22.4)

The mean numbers of different neurochemical types of CTb-labelled bouton that contacted the 18 NK1r^+^ and 20 NK1r-lacking lamina I projection neurons, together with the density of contacts (per 1000 μm^2^ of combined somatic and dendritic surface). Ranges are given in parentheses.

In contrast, the densities of contacts from both the CGRP^+^ and VGLUT2^+^/CGRP^−^ boutons that lacked CTb were considerably higher on the NK1r^+^ cells (26.6 and 16.8/1000 μm^2^, respectively) than on the NK1r-lacking cells (3.1 and 9.7/1000 μm^2^), and these differences were both significant (*P* < .001, Mann-Whitney *U* test).

**Fig. 6:**
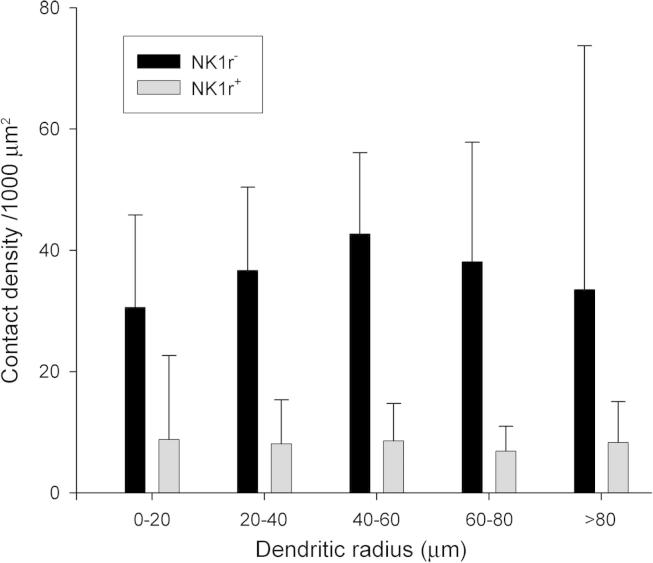
Sholl analysis of the contact densities of nonpeptidergic CTb-labelled boutons on dendrites of projection neurons. The mean densities (± standard deviation) of contacts from CTb^+^/CGRP^−^ boutons on cells without (NK1r^−^, n = 20) or with (NK1r^+^, n = 18) the NK1 receptor. For each cell, 20-μm shells were centred on the midpoint of the soma and the contact density on dendrites occurring within each shell were measured. Note that although there is considerable variability within each shell, there is no clear trend toward either increasing or decreasing density with distance from the soma for either projection neuron population.

Because the NK1r-immunostaining outlined the dendrites of NK1r^+^ projection neurons, these dendrites could be traced until they terminated or left the section. However, although Fluorogold filling allowed considerable lengths of dendrites of the NK1r-lacking projection cells to be identified, it is unlikely that these could be followed to their terminations, and therefore a greater proportion of distal dendrites will have been excluded from the analysis for the NK1r-lacking cells. If contacts were highly concentrated on the proximal dendrites of projection neurons, then the identification of more distal dendrites for the NK1r^+^ cells could have contributed to the difference in contact density that we observed for the CTb^+^/CGRP^−^ boutons. In order to test for this possibility, we performed a Sholl analysis, using shells with incremental separations of 20 μm (Fig. 6). The results of this analysis showed that although contact density varied considerably within each projection neuron population, there was no sign of clustering of contacts on proximal dendrites for either population. It is therefore unlikely that a lower density of contacts from CTb^+^/CGRP^−^ boutons on distal dendrites of the NK1r^+^ projection neurons contributed to the lower overall contact density that was seen on these cells.

### 3.3 Combined confocal and electron microscopy

**Fig. 7:**
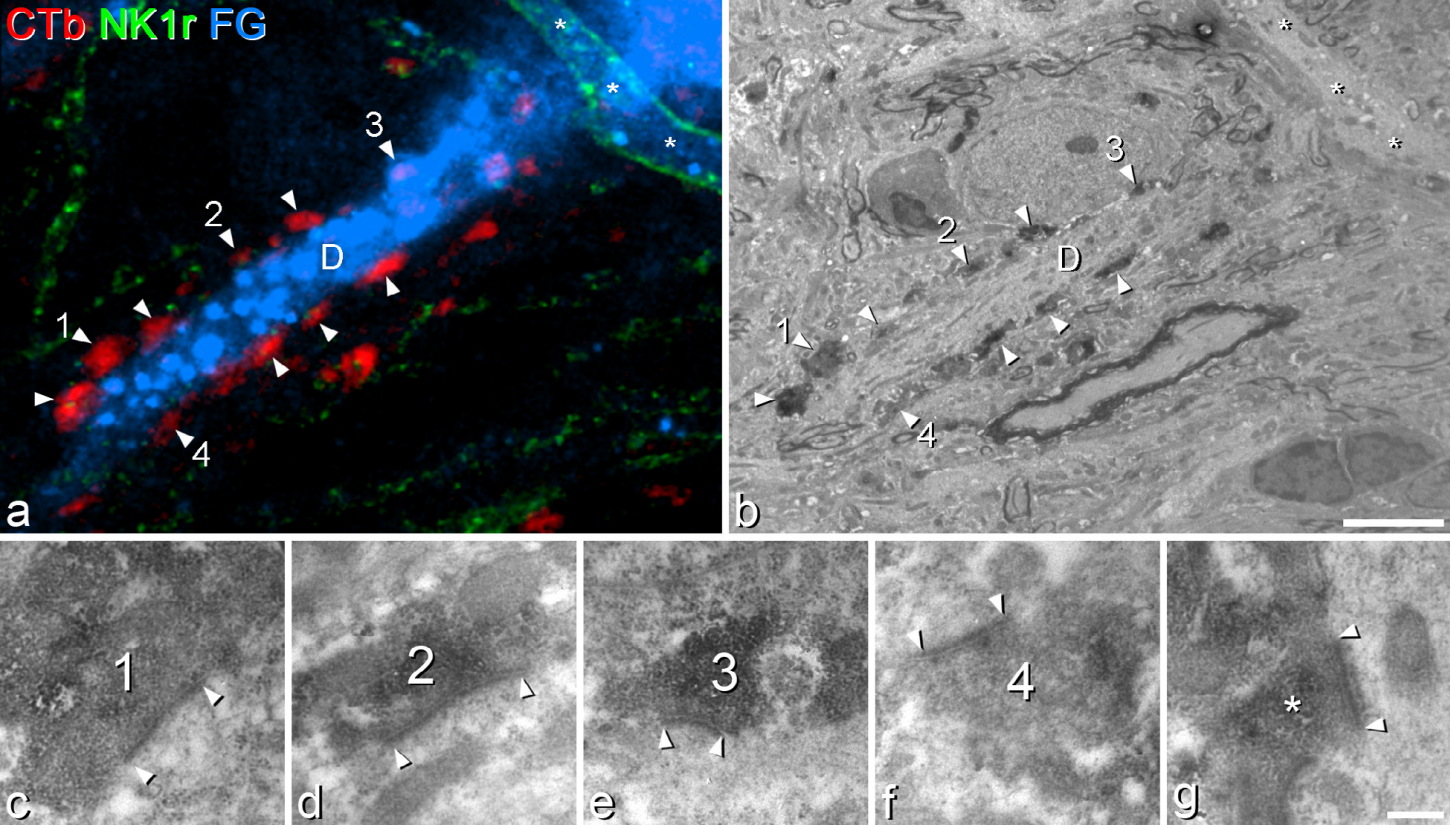
Combined confocal and electron microscopy. (a) High-magnification confocal image (single optical section) through part of a dendrite [D] of one of the NK1r-lacking spinoparabrachial lamina I cells that was analysed with combined confocal and electron microscopy. It has been scanned to reveal CTb (red), NK1 receptor (green), and Fluorogold (FG, blue). The dendrite receives numerous contacts from CTb-labelled boutons (some marked with arrowheads). The numbers correspond to boutons that are illustrated in the high-magnification electron microscope (EM) images (c–f). Note that the bouton numbered 3 is partially obscured by Fluorogold in this confocal image. A small part of a dendrite belonging to a different spinoparabrachial cell is visible in the top right corner, and is indicated with asterisks. This cell expressed the NK1 receptor, which can be seen outlining the dendrite. (b) A low-magnification EM image of the region illustrated in (a), with corresponding structures marked. (c–f) High-magnification EM images of the 4 boutons indicated in (a) and (b). In each case, the synaptic specialisation is indicated (between arrowheads). (g) One of the synapses formed by a CTb-labelled bouton (^∗^) onto a dendrite of the NK1r^+^ lamina I spinoparabrachial neuron that was analysed. Again, the synaptic specialisation is between the arrowheads. Scale bars: (b) = 5 μm [also applies to (a)]; (g) = 0.5 μm [also applies to (c-f)].

Although the ultrastructural preservation of the tissue was compromised by the lack of glutaraldehyde in the primary fixative, the CTb-labelled boutons and the dendrites of the selected projection neurons could easily be recognised (Fig. 7a and b), and synapses could be identified. We were able to find a total of 32 CTb^+^ boutons that were in contact with the 2 NK1r-lacking spinoparabrachial cells (19 on one cell and 13 on the other). In the great majority of cases (17 of 19 on the first cell and all 13 on the second cell), the bouton was seen to form an asymmetrical synapse with the projection neuron (Fig. 7c to f). Six CTb-labelled boutons in contact with the NK1r^+^ projection neuron were identified, and 5 of these were associated with an asymmetrical synapse (Fig. 7g).

## 4 Discussion

The main findings of this study are: (1) that the great majority of CTb-labelled sciatic afferents in lamina I are nonpeptidergic, and (2) that these afferents preferentially innervate a subset of NK1r-lacking projection neurons in this lamina.

### 4.1 CTb-labelling of presumed Aδ nociceptors

Although CTb can be transported by unmyelinated visceral primary afferents [45], [66] and axotomised somatic C fibres [50], [62], several lines of evidence have led to the suggestion that transport by intact somatic afferents is largely restricted to those with myelinated axons [23], [43], [44], [69]. Firstly, the great majority (94% to 97%) of neurons in the L5 dorsal root ganglion that bind CTb stain with the anti-neurofilament antibody RT97 [44], [46], which has been used as a marker for cells with myelinated axons. Secondly, the laminar distribution of labelling after sciatic injection of CTb or CTb-HRP [23], [42], [49], [50], [51], [69], [70] matches that of myelinated primary afferents [8], [28], [29], [52]. Thirdly, LaMotte et al. [23] showed that virtually all axons in the L4 dorsal root that contained CTb-HRP transported from the sciatic nerve were myelinated.

When CTb is injected into a chronically injured nerve, the pattern of central labelling changes and CTb-containing boutons appear in the outer part of lamina II (IIo). This is thought to result from transport by axotomised C fibres that have upregulated the GM1 ganglioside [5], [50], [62]. Although it is possible that some C fibres were damaged by the sciatic nerve injections in our experiments, it is very unlikely that this would have resulted in CTb transport because the change in central labelling takes longer than 3 days after injury to develop [5], [70]. Although we cannot rule out the possibility that the CTb in lamina I labels another population, such as thermoreceptors (which are thought to have unmyelinated axons in the rat [14], [30]), it is highly likely that the CTb-labelled boutons that we observed in lamina I belong to myelinated nociceptive primary afferents, and specifically those with Aδ axons [29], [67], [68].

### 4.2 Neuropeptides in Aδ nociceptors

Electrophysiological recording from dorsal root ganglion cells in several species has demonstrated that many myelinated primary afferents express neuropeptides [22], [25], [26], [27]. For example, Lawson et al. reported that 5 of 12 Aδ nociceptors in the guinea pig were CGRP-immunoreactive [25], whereas 8 of 16 contained substance P [26]. We have previously reported that approximately 20% of CTb-labelled boutons in lamina I lack detectable VGLUT2 [58], and because many peptidergic primary afferent boutons are not VGLUT2-immunoreactive [24], [32], [58], it was possible that the VGLUT2^−^/CTb^+^ boutons would correspond to peptidergic afferents. However, we found that only 11% of CTb-labelled afferents in lamina I contained CGRP, and most of these were VGLUT2-immunoreactive. Substance P was present in an even smaller proportion (2%) of the CTb-labelled boutons in this lamina.

Apart from species differences, there are 3 possible explanations for the discrepancy between the number of Aδ nociceptors found to express neuropeptides in electrophysiological studies [25], [26] and the low proportion of CTb-labelled peptidergic boutons seen in this study: (1) central terminals of peptidergic Aδ nociceptors seldom terminate in lamina I, (2) the levels of neuropeptide in their central terminals are often undetectable, or (3) most of them do not transport CTb because they lack the GM1 ganglioside. The third explanation seems most likely because Robertson and Grant [44] reported that only 3% of L5 dorsal root ganglion cells that bound CTb were substance P-immunoreactive. This interpretation is also consistent with the finding by LaMotte et al. [23] that although most large myelinated axons in the L4 dorsal root were labelled after sciatic nerve injection of CTb-HRP, label was seen in a lower proportion of fine myelinated axons.

Lawson et al. [26] reported that substance P was present in most Aδ nociceptors that responded to both noxious heat and mechanical stimulation, as well as those with deep receptive fields. However, none of the 20 Aδ high-threshold mechanoreceptors with superficial cutaneous receptive fields contained the peptide. It has since been suggested that many Aδ fibres originally classified as Aδ high-threshold mechanoreceptors [9] can respond to noxious heat, but with high thresholds (>53°C) and long latencies, and these have been classified as type I afferents [41], [65]. The almost complete lack of coexistence of substance P and CTb seen in the present study suggests that the CTb-labelled boutons in lamina I may correspond to central terminals of type I Aδ nociceptors.

### 4.3 Synaptic input from presumed Aδ nociceptors to lamina I projection neurons

Between 75% and 80% of lamina I projection neurons express the NK1r [12], [53], [59], and these are densely innervated by substance P-containing primary afferents [60]. Around one fourth of these cells were found to receive contacts from CTb-labelled afferents, most of which were nonpeptidergic, and the electron microscopic results indicate that at least some of these are associated with synapses. However, the density of CTb contacts on these cells was relatively low compared with that of peptidergic afferents. Torsney and MacDermott have investigated primary afferent input to NK1r-expressing lamina I neurons, most of which were likely to be projection cells [63], [64]. Around 30% of these received monosynaptic Aδ input, presumably from Aδ nociceptors. Interestingly, hindpaw inflammation increased the proportion of cells with monosynaptic Aδ input to approximately 60%, and this was thought to reflect activation of previously silent synapses [63]. Although the Aδ input seen in these experiments may have involved nonpeptidergic afferents, it is likely that some of the substance P^+^/CGRP^+^ boutons that synapse on NK1r-immunoreactive projection neurons [60] belong to peptidergic Aδ nociceptors, and that these contributed to the monosynaptic input.

Giant lamina I projection neurons have a highly characteristic pattern of inhibitory and excitatory synaptic input [35], [40]. However, these cells are rare, accounting for approximately 3% of lamina I projection neurons. Therefore the remaining NK1r-lacking neurons constitute approximately 20% of the projection cells in this lamina. Until now, little was known about their synaptic inputs, but here we show that >40% of them receive contacts from presumed nonpeptidergic Aδ nociceptors, that these contacts can be extremely numerous, and that they are associated with synapses.

Excitatory synapses in lamina I originate from local neurons, primary afferents, and descending axons [57]. The VGLUT2 antibody is likely to reveal the axons of all local excitatory neurons [58], [71], as well as any descending axons, except for corticospinal axons, which are sparse in this lamina [13]. Apart from Aδ nociceptors, most primary afferents in lamina I are peptidergic C fibres, which are CGRP-immunoreactive [19]. However, there is also a population of nonpeptidergic TRPM8^+^ thermoreceptive C fibres [11], and it is not yet known whether these express VGLUT2. It is therefore likely that most other glutamatergic boutons in lamina I (with the possible exception of TRPM8^+^ afferents) would be detected by the combination of CGRP and VGLUT2 antibodies used in this study. The mean density of contacts from CTb^+^/CGRP^−^ boutons on the 20 NK1r-lacking projection neurons was 32/1000 μm^2^, whereas the mean density of contacts from all other immunostained boutons (ie, those with CGRP and/or VGLUT2) was 13/1000 μm^2^ (Table 4). For this group of NK1r-lacking projection cells, nonpeptidergic Aδ (type I [41], [65]) nociceptors could therefore provide up to 70% of their excitatory synapses, suggesting a very powerful synaptic input.

Two studies have tested responses of lamina I spinoparabrachial neurons to mechanical and thermal stimuli. All 53 cells recorded by Bester et al. [7] were activated by noxious heat, although with varying thresholds, and 92% responded to noxious mechanical stimuli. Andrew [3] also found that approximately 95% of spinoparabrachial neurons were driven by both noxious heat and mechanical stimuli. This indicates that the vast majority of lamina I spinoparabrachial neurons respond to noxious stimuli, and these presumably include most of the NK1r-lacking cells. Interestingly, a study using Fos as an activation marker [18] reported that NK1r-lacking lamina I projection neurons were significantly less likely to show Fos than those with the receptor after brief immersion of the foot in water at 52°C. This stimulus evoked Fos in 63% of NK1r^+^ lamina I spinoparabrachial cells, but only in 14% of those without the receptor [61]. The discrepancy between this result and the reports that virtually all spinoparabrachial lamina I cells respond to noxious heat [3], [7] may be because nonpeptidergic Aδ nociceptors with high heat thresholds (>53°C) [41], [65] innervate many of the NK1r-lacking cells.

The finding that over 40% of NK1r-lacking spinoparabrachial cells are innervated by presumed Aδ nociceptors suggests that these cells have an important role in perception of fast pain. Ablation of NK1r^+^ lamina I neurons with substance P-saporin reduced hyperalgesia in chronic pain states, but left acute pain thresholds intact [31], [54]. Our findings suggest that synaptic input from Aδ nociceptors to NK1r-lacking lamina I projection cells may have played a role in maintaining acute nociception in these animals.

These results provide further evidence that the primary afferent input to different types of projection neuron is organised in a specific way. This indicates that functional differences between nociceptor subtypes are, to some extent, maintained at the level of the projection neurons, which form the major output from the superficial dorsal horn.

## Conflict of interest statement

The authors report no conflicts of interest.
